# Characteristics of Smoothing Filters to Achieve the Guideline Recommended Positron Emission Tomography Image without Harmonization

**DOI:** 10.22038/aojnmb.2017.26684.1186

**Published:** 2018

**Authors:** Yuji Tsutsui, Shinichi Awamoto, Kazuhiko Himuro, Yoshiyuki Umezu, Shingo Baba, Masayuki Sasaki

**Affiliations:** 1 Division of Radiology, Department of Medical Technology, Kyushu University Hospital, Fukuoka, Japan; 2 Medical Quantum Science Course, Department of Health Sciences, Graduate School of Medical Sciences, Kyushu University, Fukuoka, Japan; 3 Department of Clinical Radiology, Graduate School of Medical Sciences, Kyushu University, Fukuoka, Japan; 4 Department of Health Sciences, Faculty of Medical Sciences, Kyushu University, Fukuoka, Japan

**Keywords:** FDG PET, Smoothing filter, SUV

## Abstract

**Objective(s):**

The aim of this study is to examine the effect of different smoothing filters on the image quality and SUV_max_ to achieve the guideline recommended positron emission tomography (PET) image without harmonization.

**Methods:**

We used a Biograph mCT PET scanner. A National Electrical Manufacturers Association (NEMA) the International Electrotechnical Commission (IEC) body phantom was filled with ^18^F solution with a background activity of 2.65 kBq/mL and a sphere-to-background ratio of 4. PET images obtained with the Biograph mCT PET scanner were reconstructed using the ordered subsets-expectation maximization (OSEM) algorithm with time-of-flight (TOF) models (iteration, 2; subset, 21); smoothing filters including the Gaussian, Butterworth, Hamming, Hann, Parzen, and Shepp-Logan filters with various full width at half maximum (FWHM) values (1-15 mm) were applied. The image quality was physically assessed according to the percent contrast (Q_H,10_), background variability (N_10_), standardized uptake value (SUV), and recovery coefficient (RC). The results were compared with the guideline recommended range proposed by the Japanese Society of Nuclear Medicine and the Japanese Society of Nuclear Medicine Technology. The PET digital phantom was developed from the digital reference object (DRO) of the NEMA IEC body phantom smoothed using a Gaussian filter with a 10-mm FWHM and defined as the reference image. The difference in the SUV between the PET image and the reference image was evaluated according to the root mean squared error (RMSE).

**Results:**

The FWHMs of the Gaussian, Butterworth, Hamming, Hann, Parzen, and Shepp-Logan filters that satisfied the image quality of the FDG-PET/CT standardization guideline criteria were 8-12 mm, 9-11 mm, 9-13 mm, 10-13 mm, 9-11 mm, and 12-15 mm, respectively. The FWHMs of the Gaussian, Butterworth, Hamming, Hann, Parzen, and Shepp-Logan filters that provided the smallest RMSE between the PET images and the 3D digital phantom were 7 mm, 8 mm, 8 mm, 8 mm, 7 mm, and 11 mm, respectively.

**Conclusion:**

The suitable FWHM for image quality or SUV_max_ depends on the type of smoothing filter that is applied.

## Introduction

^18^F-2-fluoro-2-deoxy-D-glucose (^18^F-FDG) positron emission tomography/computed tomography (PET/CT) is widely used for the diagnosis and staging of various malignancies and is a valuable tool for the assessment of the response to therapy (1-5). The standardized uptake value (SUV), which is a semi-quantitative metric of FDG-PET, is thought to be helpful for diagnoses, prognostic stratification, and therapy monitoring. Because SUV assesses metabolic information, it could be a better biomarker for treatment responses than anatomical morphological information (6-9).

SUV is the standard semi-quantitative measure derived from whole-body FDG PET/CT examinations; therefore, repeatability and reproducibility are required for it to be used as a biomarker. The advancement of PET/CT scanners has resulted in wide variations in their basic performance. There are considerable differences in SUV due to the PET scanner characteristics, acquisition settings, reconstruction algorithms, and settings (10, 11). Different examination protocols are another cause of SUV variation. Different data acquisition and processing protocols result in variations in the SUV measurements (10-13). Several academic societies have published FDG-PET examination guidelines for standardization (14, 15). However, variations in SUV still exist. For example, a point spread function (PSF) algorithm can improve the spatial resolution even though it causes edge artifacts as an overshoot (16).

The concept of harmonization was developed to directly compare the SUV values between different institutions and devices. As part of harmonizing the PET/CT performance, several organizations, including the American College of Radiology, the Society of Nuclear Medicine Clinical Trials Network (SNM-CTN), and the European Association of Nuclear Medicine (EANM), have set up PET/CT validation procedures as part of site accreditation for multicenter studies. The representative harmonization is to adjust the RC of each PET scanner into the reference RC range using an additional Gaussian filter (GF). FDG-PET harmonization is reported to minimize the variation in SUV measurements via scan acquisition and processing (17, 18). Although the application of GF on reconstructed image file is used as harmonization, the smoothing filter used in the reconstruction process is considered to result the similar effect. However, there are few studies demonstrating the characteristics of different smoothing filters on the image quality and the SUV_max_.

The purpose of this study was to evaluate the effect of different smoothing filters on the FDG-PET images. We evaluated the image quality and the SUV_max_ in comparison to the standard PET imaging protocols and phantom test procedures and criteria proposed Japanese Society of Nuclear Medicine (JSNM).

## Methods

### NEMA IEC Body Phantom

The NEMA IEC body phantom (Data Spectrum Corp., Hillsborough, NC), consisting of a quasicylindrical cavity (280×210×180 mm) with six spheres (Model ECT/IEC -BODY/P), was used for this study. The spheres were 10, 13, 17, 22, 28, and 37 mm in diameter, with a wall thickness of 1 mm. All of the spheres were filled with 10.6 kBq/mL of ^18^F-FDG, and the background was filled with 2.65 kBq/mL of ^18^F-FDG to obtain a sphere-to-background ratio of 4. The radioactivities of three 1.0 mL samples of both hot spheres and the background ^18^F-FDG were prepared for measurement using a well counter of the AccuFLEX γ7001 (Hitachi, Ltd., Tokyo, Japan). The radioactivity of ^18^F-FDG was corrected for decay with the ^18^F half-life of 109 min.

### PET/CT Scanner

The PET/CT data were acquired using a Biograph mCT (Siemens Healthineers, Erlangen, Germany). This PET scanner has three rings each containing 144 lutetium orthosilicate of 4×4×20 mm. The axial field of view (FOV) was 16.2 cm, and the transaxial FOV was 70 cm in diameter. The coincidence time window was 4.1 ns, and the spatial resolutions at 1 cm and 10 cm were 4.4 mm and 4.9 mm full width at half maximum (FWHM), respectively. The system sensitivities when the line source was at 0 cm and 10 cm from the center of the FOV were 0.96% and 0.94%, respectively.

### Digital phantom

The PET digital phantom developed from the PET digital reference object (DRO) is based on a modified version of the NEMA Image Quality Phantom. The NEMA phantom and the digital object were mathematically developed as an ideal object with a uniform background region that is 20 cm in the transaxial simulated human abdominal cross section with an SUV of 1.00. There are six spheres, whose diameters are 10, 13, 17, 22, 28, and 37 mm with an SUV of 4.00 and a central 5 cm diameter cylinder with an SUV of 0.00 (19). DRO was applied a 3-D GF to simulate the PET image with relatively decreased spatial resolution. The RC of DRO was included in the JSNM reference range with a GF of 10-13 mm FWHM, and the highest RC was obtained with a 10 mm FWHM of GF. DRO_10mm_ was adopted as reference SUV for the evaluation.

### Data acquisition and processing

The emission data were acquired for 3 min for the evaluation of the image quality and for 30 min to determine the parameters for the evaluation of the SUV_max_. The PET images were reconstructed using 3D ordered subsets-expectation maximization (OSEM) with a time-of-flight (TOF) algorithm with CT attenuation correction. PSF correction was not used for the reconstruction. The reconstruction parameters were as follows: a pixel size of 3.18×3.18 mm; 2 iterations; 21 subsets; and a slice thickness of 5.0 mm. The CT scanning parameters were as follows: 120 kV, 100 mAs (Eff.mAs), a 512×512 matrix, 32-slices, a slice thickness of 3.0 mm, and a 500-mm transaxial FOV.

Six smoothing filters including the Gaussian, Butterworth, Hamming, Hann, Parzen, and Shepp-Logan filters were applied (Table 1). The GF was used to smooth the image with a Gaussian function. With a pixel size of Δx in mm, the filter width is *2b+1* pixels with

**Table 1:** The definition of each smoothing filters including Gaussian, Butterworth, Hamming, Hann, Parzen and Shepp-Logan filters.

Smoothing filter	The definition	
Gaussian	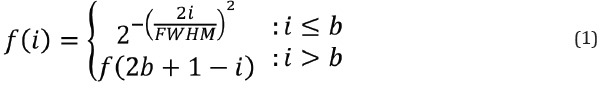	(1)
Butterworth	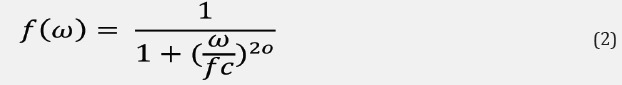	(2)
Hamming	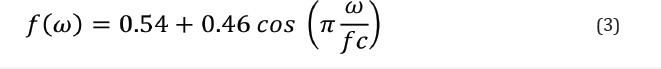	(3)
Hann	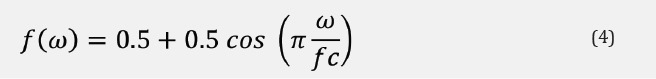	(4)
Parzen	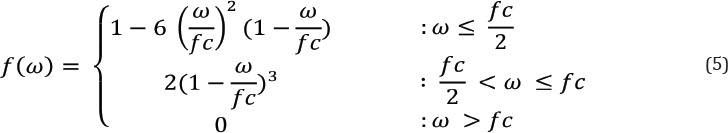	(5)
Shepp-Logan	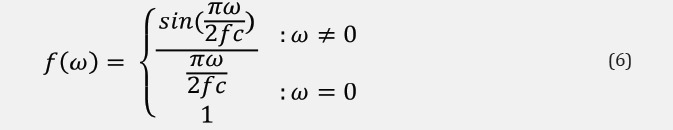	(6)


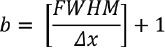


The filter was symmetrical and was calculated using equation (1). The filter width given in the FWHM needs to be converted into the cut-off frequency *fc*. The cut-off frequency is a fraction of the Nyquist frequency, which is defined as half of the sampling frequency , where Δx is the sampling distance in mm. The Butterworth filter is defined by equation (2) with a frequency ω and a filter order *o*. The Hamming filter is defined by equation (3) with a frequency ω. The Hann filter is defined by equation (4) with a frequency ω. The Parzen filter is defined by equation (5) with a frequency ω. The Shepp-Logan filter is defined by equation (6) with the frequency ω. The FWHM of the smoothing filters ranged from 1 mm to 15 mm, the order of the Butterworth filter was 1.0.

### Analysis of the Image quality

The region of interest (ROI) for a 10-mm sphere was a circular ROI with a diameter of 10 mm placed on the cross section through the center of the sphere. We placed twelve 10-mm circular ROIs for the background on five slices. We also placed a volume of interest for all hot spheres equal to each sphere’s inner diameter. The PET images were analyzed using *Q_H,10mm_* for percent contrast, *N_10mm_* for the background variability, and SUV as the standardized uptake value.

These parameters were calculated using the following equations:


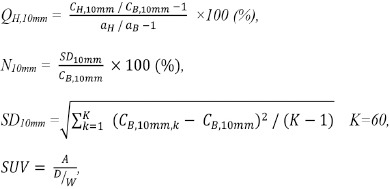


where *C_H,10mm_* is the average count in the ROI for a 10-mm sphere, *C_B,10mm_* is the average count of the 60 background ROIs, *a_H_* and *a_B_* are the true radioactivity concentrations in the hot sphere and background, respectively. *SD_10mm_* is the standard deviation of the 60 background ROIs, A is the measured activity in ROI (Bq/mL), D is the injected dose (Bq), and W is the body weight (g).

**Figure 1:**
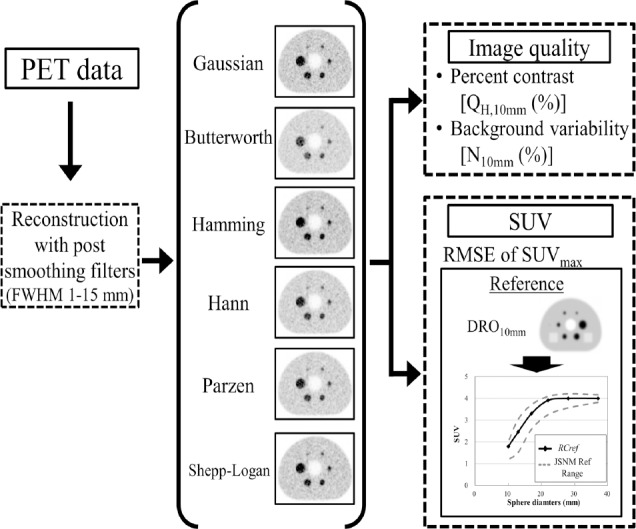
Flow chart for the evaluation of the image quality and harmonization for different smoothing filters and FWHMs.

The image quality was evaluated and compared with the reference value in the “Japanese Guideline for Oncology FDG PET/CT Data Acquisition Protocol: Synopsis of Version 2.0” published by the JSNM and the Japanese Society of Nuclear Medicine Technology (JSNMT) (20). The guideline recommended N_10mm_<5.6 (%) as a background noise level and Q_H,10mm_ / N_10mm_ >2.8 (%) as a contrast noise ratio to obtain standardized PET image. SUV_max_ was plotted as a function of the sphere diameter and compared to the reference range proposed by JSNM. The reference ranges of the lower-upper limits of SUV_max_ on the 10, 17, 22, 28, and 37-mm hot spheres are 1.19-2.00, 1.52-3.04, 2.58-3.71, 3.25-4.09, 3.56-4.21 and 3.82-4.17, respectively (Figure 1).

### Analysis of the SUV_max_

**Figure 2:**
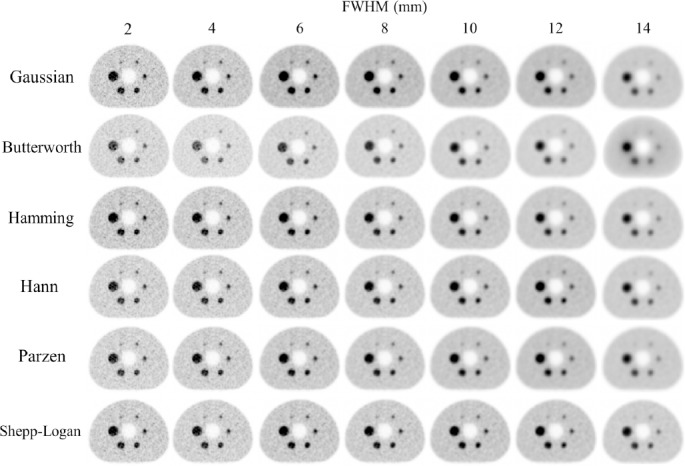
PET images of the NEMA IEC Body phantom using different smoothing filters and FWHMs.


To evaluate the SUV_max_, we defined the SUV of the digital body phantom as the reference SUV (SUVref_j_: j=sphere diameter) and the SUV of the PET scanner as the target SUV (SUV_j_). We used the root mean squared error (RMSE) of the SUV to evaluate the difference between the target and the reference SUV (Figure 2).

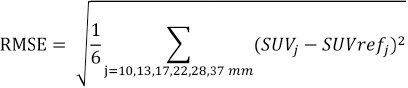


## Results

### PET images

Figure 2 shows the PET images of the NEMA phantom using different filters and the FHWM. With increasing of FWHM, the images became blurred and the noise decreased for all smoothing filters. However, the influence of the FWHM on the smoothing differed between filters.

### Image quality

**Figure 3:**
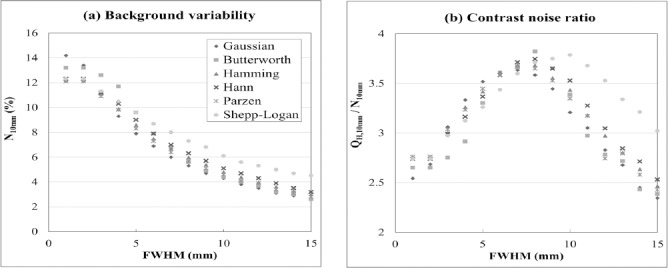
The image quality in relation to the different smoothing filters and FWHMs: (a) background variability; (b) Contrast noise ratio.

Figure 3 shows the effect of the smoothing filters on the image quality in relation to FWHM. For all smoothing filters, N_10mm_ decreased with increasing of FWHM. However, the effect of the FWHM on the image quality varied in relation to the type of smoothing filter (Figure 3a). The FWHM that satisfied the recommended values proposed by the FDG-PET/CT standardization guideline criteria (N_10mm_<5.6 (%) and Q_H,10mm_/N_10mm_ >2.8 (%)) and supplied the maximum Q_H,10 mm_/N_10 mm_ differed between the smoothing filters (Figure 3a and 3b). The FWHMs of the Gaussian, Butterworth, Hamming, Hann, Parzen, and Shepp-Logan filters that satisfied the image quality of the FDG-PET/CT standardization guideline criteria were 8-12 mm, 9-11 mm, 9-13 mm, 10-13 mm, 9-11 mm, and 12-15 mm, respectively (Table 2).

**Table 2:** The recommended FWHM of smoothing filters determined by imaging quality and the SUV_max_.

		Gaussian	Butterworth	Hamming	Hann	Parzen	Shepp-Logan
**Image quality**	N_10mm_ < 5.6	> 8	> 9	> 9	> 10	> 9	> 12
	Q_H,10mm_ / N_10mm_ > 2.8	8-12	9-11	9-13	10-13	9-11	12-15

**SUV_max_**	JSNM Ref Range	6-10	7-10	7-11	7-12	7-10	9-15
	The smallest RMSE	7	8	8	8	7	11

### SUV_max_

**Figure 4:**
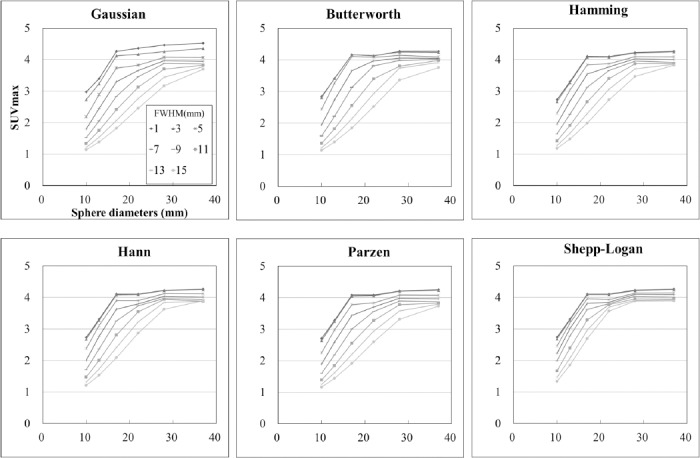
Recovery coefficients in relation to the different smoothing filters and FWHMs.

Figure 4 shows SUV_max_ in relation to the sphere diameters according to the different FWHMs of the smoothing filters. For all the smoothing filters, SUV decreased with increasing of FWHM. However, the influence of the FWHM on SUV_max_ differed between the smoothing filters. The FWHMs that permitted SUV_max_ to be included in the JSNM reference range differed between the smoothing filters, and the FWHM ranges of the Gaussian, Butterworth, Hamming, Hann, Parzen, and Shepp-Logan filters were 6-10 mm, 7-10 mm, 7-11 mm, 7-12 mm, 7-10 mm, and 9-15 mm, respectively (Table 2).

**Figure 5:**
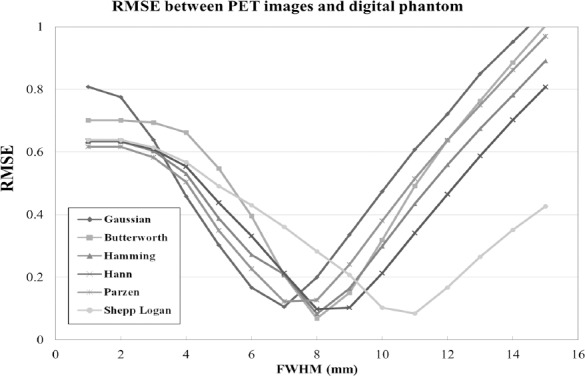
RMSE between the PET images with different smoothing filters and the digital phantom.

**Figure 6:**
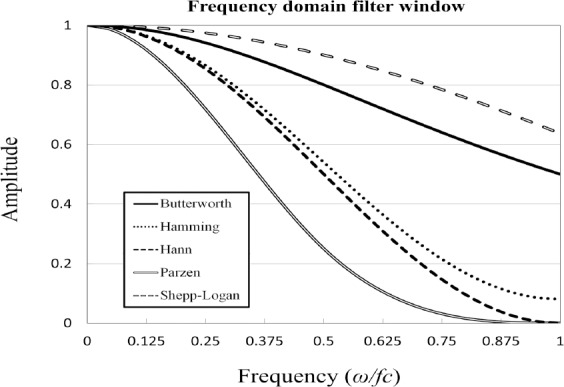
Frequency amplitude in relation to the frequency of the different smoothing filters.

Figure 5 shows the RMSE of the six smoothing filters in relation to the FWHM. The FWHM that provided the smallest RMSE differed between the smoothing filters. The FWHM values of the Gaussian, Butterworth, Hamming, Hann, Parzen, and Shepp-Logan filters that provided the smallest RMSE were 7 mm, 8 mm, 8 mm, 8 mm, 7 mm, and 11 mm, respectively (Table 2). Figure 6 shows the relationship between the amplitude and the frequency for the frequency domain filters created from the equation (2-6). The Parzen filter showed the highest smoothing followed by the Hann, Hamming, Butterworth, and Shepp-Logan filters.

## Discussion

We examined the effect of six smoothing filters on the image quality and the SUV_max_ of FDG-PET images. The effect on both the image quality and SUV_max_ differed between the different smoothing filters. The suitable FWHM to obtain adequate image quality and SUV_max_ depends on the type of smoothing filter.

Both the percent contrast and the background variability decreased with increasing of FWHM for all smoothing filters. However, the relationship between the percent contrast and the background variability is similar for the different smoothing filters. Tong et al. evaluated the noise and signal properties for different combinations of reconstruction methods and parameters including no-filter, 4, 7, and 10-mm GF. They reported that the background variability and contrast recovery decreased with increasing post-filtering (21). Alessio et al. evaluated the image quality and noise resolution for different smoothing parameters of PET images. They reported that the post-reconstruction filter varied changing the noise/bias tradeoff (22).

In this study, SUV decreased with the increasing FWHM, irrespective of the type of smoothing filter. Westerterp et al. evaluated the effect of the image reconstruction, resolution, and ROI definition parameters for the FDG-PET quantification. They also reported that SUV decreased with the increasing of FWHM of the post-filter using GF of 5, 7, and 9-mm FWHM (11). However, the degree of SUV change in relation to the FWHM was dependent on the type of smoothing filter.

The suitable FWHM of the GF for SUV_max_ was 7.0 mm in our study. The EANM guideline reported that spatial filters applied during or after reconstruction should not exceed a FWHM of 7 mm (14). Saha reported that the largest amount of smoothing is provided by the Parzen filter, while the Shepp-Logan filter produces the least smoothing (23). In this study, the FWHMs of the Butterworth, Hamming, Hann, Parzen, and Shepp-Logan filters that provided the smallest RMSE between the SUVs of the PET image and the digital phantom were 8 mm, 8 mm, 8 mm, 7 mm, and 11 mm, respectively. To adjust the RC for same reference RC, a high smoothing filter requires a small FWHM and a low smoothing filter requires a large FWHM. Regarding all smoothing filters, the suitable FWHM varied between filters. The FWHM functions differently in each filter due to differences in the characteristics of the frequency. It is necessary to determine the suitable FWHM for each filter.

This study has some limitations. First, the digital phantom, which was developed from a CT image of an NEMA body phantom obtained with a GF with a 10-mm FWHM, was defined as the reference for the SUV_max_. It is thought that the suitable FWHM of the smoothing filter may vary for other references of SUV_max_. Second, the PET images were reconstructed using 3D OSEM with a TOF algorithm without PSF correction. The suitable FWHM for SUV_max_ may vary due to the reconstruction parameters. Finally, we evaluated SUV relation to the different smoothing filters using only SUV_max_, therefore further examination is required to examine the SUV_peak_ and SUV_mean_.

## Conclusion

The suitable FWHM for image quality or SUV_max_ depends on the smoothing filter. Each smoothing filter could provide SUV_max_ that satisfy the JSNM reference range with its own suitable FWHM.
